# Diagnostic value of dual-tracer PET/CT with [^18^F]FDG and PSMA ligands in prostate cancer: an updated systematic review

**DOI:** 10.3389/fmed.2025.1607227

**Published:** 2025-07-09

**Authors:** Cesare Michele Iacovitti, Marco Cuzzocrea, Alessio Rizzo, Matteo Bauckneht, Roberto C. Delgado Bolton, Gaetano Paone, Giorgio Treglia

**Affiliations:** ^1^Division of Nuclear Medicine, Imaging Institute of Southern Switzerland, Ente Ospedaliero Cantonale, Bellinzona and Lugano, Switzerland; ^2^Division of Nuclear Medicine, Candiolo Cancer Institute, Turin, Italy; ^3^IRCCS Ospedale Policlinico San Martino, Genoa, Italy; ^4^Department of Health Sciences (DISSAL), University of Genoa, Italy; ^5^Department of Diagnostic Imaging (Radiology) and Nuclear Medicine, University Hospital San Pedro and Centre for Biomedical Research of La Rioja (CIBIR), Logroño, La Rioja, Spain; ^6^Servicio Cántabro de Salud, Santander, España; ^7^Faculty of Biomedical Sciences, Università della Svizzera italiana, Lugano, Switzerland; ^8^Faculty of Biology and Medicine, University of Lausanne, Lausanne, Switzerland

**Keywords:** prostate cancer, PSMA, [^18^F]FDG, PET/CT, dual-tracer, hybrid imaging, diagnostic, Gleason score

## Abstract

**Background:**

Prostate-specific membrane antigen (PSMA) ligand PET/CT has significantly improved prostate cancer (PCa) imaging. However, in patients with poorly differentiated PCa or neuroendocrine transdifferentiation, [^18^F]fluorodeoxyglucose ([^18^F]FDG) PET/CT may provide additional diagnostic information. This systematic review evaluates the diagnostic value of combining [^18^F]FDG PET/CT with PSMA ligand PET/CT in PCa patients.

**Methods:**

A systematic literature search of studies assessing the added diagnostic value of dual-tracer [^18^F]FDG and PSMA ligands PET/CT in PCa patients was conducted using PubMed/MEDLINE and Cochrane Library databases and available information was summarized.

**Results:**

Fourteen studies (*n* = 901 patients) met the inclusion criteria. The dual-tracer approach identified [^18^F]FDG-positive/PSMA-negative (FDG+/PSMA−) lesions in a subset of patients, particularly those with Gleason Score (GS) ≥ 9. However, in patients with GS < 8, [^18^F]FDG PET/CT did not significantly improve lesion detection over PSMA ligand PET/CT alone.

The presence of FDG+/PSMA− lesions correlated with aggressive tumor biology, increased risk of metastases, and worse prognosis.

**Conclusion:**

Literature data showed that [^18^F]FDG PET/CT may serve as a valuable complementary imaging modality for high risk PCa patients potentially influencing staging and treatment decisions. Future prospective studies are warranted to further elucidate the prognostic significance and cost-effectiveness of combining [^18^F]FDG PET/CT with PSMA ligand PET/CT in PCa patients.

## 1 Introduction

Prostate cancer (PCa) represents the third most frequently diagnosed malignancy worldwide (1, 2). This high prevalence highlights the importance of improving diagnostic and therapeutic approaches to manage the disease effectively.

Metastatic PCa is initially hormone-sensitive; however, over time, it can acquire resistance to therapy, leading to the development of metastatic castration-resistant prostate cancer (mCRPC) (3).

This progression represents a significant clinical challenge, making it crucial to accurately identify the locations and extent of the disease to guide appropriate therapeutic strategies.

Since their introduction, prostate-specific membrane antigen (PSMA) ligand positron emission tomography/computed tomography (PET/CT) has markedly enhanced imaging sensitivity in PCa (4–6).

Although PSMA ligand PET/CT is generally considered superior to fluorine-18 fluorodeoxyglucose ([^18^F]FDG) PET/CT for detecting PCa lesions (7, 8), evidence from some studies suggests that [^18^F]FDG PET/CT may have utility or even demonstrates higher diagnostic sensitivity in patients with poorly differentiated adenocarcinoma or neuroendocrine differentiation compared to those with well-differentiated adenocarcinoma (9–11).

This review aims to summarize the existing evidence on the use of dual-tracer PET, combining [^18^F]FDG PET/CT and PSMA ligand PET/CT, for staging PCa at initial diagnosis and in a recurrence/progression setting.

## 2 Materials and methods

### 2.1 Protocol

The present systematic review was conducted following a predefined protocol (12), and the “Preferred Reporting Items for a Systematic Review and Meta-Analysis” (PRISMA 2020 statement) was used as a benchmark in its production (13).

The protocol for this systematic review was not pre-registered (as permitted by item 24 of the PRISMA checklist).

The first step in this systematic review was to define the research question using the PICO framework (Population, Intervention, Comparator, Outcomes). The focus was on patients diagnosed with PCa (Population) who underwent PSMA ligand PET/CT imaging (Intervention), with the addition of [^18^F]FDG PET/CT (Comparator). The main outcomes assessed were the potential changes in diagnostic accuracy, staging, and management of PCa when [^18^F]FDG PET/CT is included alongside PSMA ligand PET/CT imaging. Two independent reviewers (G.T. and C.M.I.) conducted the literature search, study selection, and quality assessment. Any discrepancies between reviewers were resolved through a consensus meeting with a third reviewer (A.R.).

### 2.2 Literature search strategy, information sources, and eligibility criteria

After formulating the review question, two independent reviewers (G.T. and C.M.I.) conducted a systematic search of PubMed/MEDLINE and the Cochrane Library to identify studies evaluating the diagnostic value of dual-tracer [^18^F]FDG and PSMA ligand PET/CT imaging in PCa. The search strategy incorporated terms such as “PSMA” and “FDG”, tailored to the context of dual-tracer imaging approaches. Boolean operators (AND, OR) were employed to refine the search and ensure relevant studies were included. No restrictions were applied regarding the publication year. Additionally, the reference lists of all included studies were screened for potentially relevant articles. The search was last updated on October 8, 2024.

Reviews, letters, comments, editorials and case reports were excluded from the systematic review to ensure methodological rigor. Only studies published in English were considered eligible for inclusion in the review. We aimed to summarize the available evidence for dual-tracer PET with [^18^F]FDG and PSMA ligands for PCa staging and restaging. Studies using dual-tracer PET for the purpose of radioligand therapy were excluded because they were outside the scope of this systematic review.

### 2.3 Study selection process and data collection

The titles and abstracts of all retrieved articles were independently screened by two reviewers (G.T. and C.M.I.) to assess eligibility based on predefined inclusion and exclusion criteria. Discrepancies were resolved through discussion. Relevant studies were selected systematically, and data extraction was performed using a standardized form to ensure consistency and accuracy. Information was collected from full texts, tables, figures, and supplementary materials.

For each included study, the extracted data encompassed: general study details (authors, publication year, country, study design, and funding sources); patient characteristics (sample size, enrollment date, age, clinical setting, Gleason score [GS], prostate-specific antigen [PSA] level and PSA doubling-time and previous treatment); key characteristics of the index test (type of PET tracers, two-scan interval, administered activity of each radiopharmaceutical, uptake times, scan time per bed, image analysis, image evaluators and other PET tracers, when reported); and outcomes of the included studies (validation of PET findings, detection rate of [^18^F]FDG PET/CT compared to PSMA ligand PET/CT, correlation of [^18^F]FDG PET/CT findings with GS and PSA levels, additional diagnostic value by using [^18^F]FDG PET/CT and main findings).

### 2.4 Quality assessment (risk of bias assessment)

The National Institutes of Health (NIH) quality assessment tool was used to evaluate the quality of the included studies and the risk of bias. Two reviewers independently evaluated the methodological quality of the studies (G.T. and A.R.). Reviewer disagreements regarding the quality assessment were resolved through a consensus meeting.

## 3 Results

### 3.1 Literature search and study selection

**FIGURE 1:**
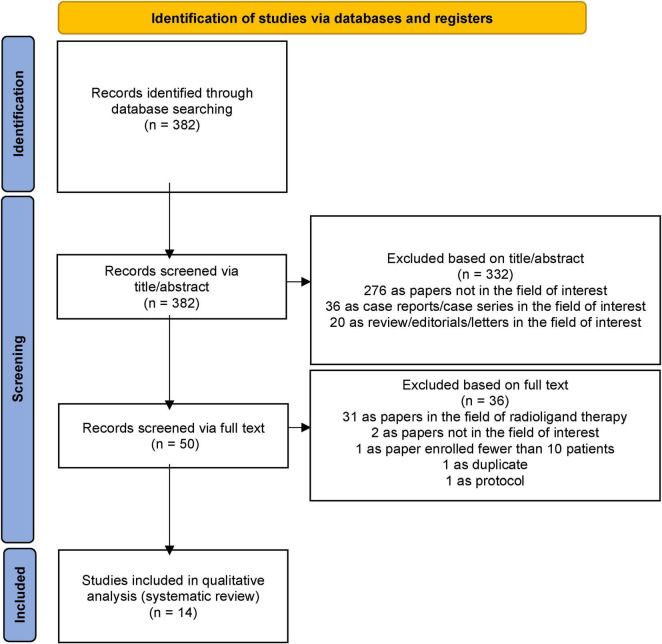
PRISMA flowchart summarizing the study selection process. PRISMA: Preferred Reporting Items for Systematic Reviews and Meta-Analysis.

The literature search resulted in the identification of 382 records, which were assessed for eligibility according to the criteria described in the Materials and methods section. Based on predefined inclusion and exclusion criteria, 332 records were excluded because they were either unrelated to the field of interest or classified as case reports/case series or reviews/editorials/letters, even if relevant to the field. Of the remaining 50 studies, 36 were excluded after an in-depth evaluation of the full text, leaving 14 studies deemed eligible for inclusion in this systematic review. The selection process is summarized in Figure 1.

*Research question*: What is the diagnostic value of adding [^18^F]FDG PET/CT to PSMA ligand PET/CT in the diagnostic pathway of patients with prostate cancer.

*Research string*: ((FDG) OR (fluorodeoxyglucose)) AND ((PSMA) OR (prostate specific membrane antigen)) AND (prostat*).

*Database screened*: PubMed/MEDLINE and the Cochrane Library.

### 3.2 Study characteristics

The 14 studies selected based on predefined inclusion and exclusion criteria comprise a total of 901 prostate cancer patients who underwent both PSMA ligand and [^18^F]FDG PET/CT imaging. Their characteristics are analyzed in detail in Tables 1–3. The studies were published between 2020 and 2024 across various countries [China (9/14), Canada (1/14), Finland (1/14), Germany (1/14), South Korea (1/14), and the USA (1/14)]. Regarding study design, 13 out of 14 were single-center studies, while one was multicenter (14). Furthermore, seven studies were prospective, and the remaining seven were retrospective. All studies provide information on funding within the text.

**TABLE 1:** General study information.

References	Publication year	Country	Study design/number of involved centers	Funding sources
Pouliot et al. (14)	2024	Canada	Multicenter (5 Canadian academic hospital centers)/prospective	ONCOPOLE EMC2
Pabst et al. (15)	2024	Germany	Single center/retrospective	Several funding sources disclosed
Pan et al. (16)	2024	China	Single center/prospective	Several funding sources disclosed
Xu et al. (17)	2023	China	Single center/retrospective	National Key R&D Programof China National Natural Science Foundation of China
Kim et al. (18)	2023	South Korea	Single center/prospective	National Research Foundation of Korea
Malaspina et al. (19)	2022	Finland	Single center/prospective	University of Turku (UTU) Turku University Central Hospital
Pan et al. (20)	2022	China	Single center/prospective	National Natural Science Foundation of China Beijing Xisike Clinical Oncology Research Foundation
Chen et al. (21)	2021	China	Single center/retrospective	National Natural Science Foundation of China
Fourquet et al. (22)	2021	Maryland (USA)	Single center/prospective	National Cancer Institute National Institutes of Health
Shi et al. (23)	2021	China	Single center/retrospective	National Natural Science Foundation of China Shanghai Advanced Appropriate Technology Promotion Projects JiangXi Provincial Department of Science and Technology Jiangxi Provincial Health Commission
Shi et al. (24)	2021	China	Single center/retrospective	National Natural Science Foundation of China
Zhou et al. (25)	2021	China	Single center/retrospective	Sichuan Science and Technology Program Health research project of Sichuan Province
Chen et al. (26)	2021	China	Single center/retrospective	National Natural Science Foundation of China
Wang et al. (27)	2020	China	Single center/prospective	Several funding sources disclosed

**TABLE 2:** Patient key characteristics and clinical settings.

References	Sample Size (No. of patients performing PSMA and FDG PET)	Enrollment date	Mean/median age (Years)	Clinical setting	GS	PSA (ng/mL)	PSA dt (months)	Previous treatment
Pouliot et al. (14)	98	NR	Mean ± SD: 69.2 ± 7.4	mCRPC	GS ≤ 6 = 4 GS 7a = 5 GS 7b = 12 GS 8 = 23 GS 9–10 = 48	Median (range): 51.1 (18.9–206.0)	NR	ADT: 98 ARPI: 87 Taxanes: 67 Radium-223: 16 PARP-I: 6 Other: 11
Pabst et al. (15)	10	August 2021 – November 2021	Median (range): 71 (62–86)	mCRPC	GS 7b = 1 GS 8 = 5 GS 9 = 3 Not available = 1	Median (range): 156 (2.5–747)	NR	ADT: 10 ARPI: 10 Taxanes: 10 Radium–223: 1
Pan et al. (16)	33	July 2020 – February 2021	Median (IQR): 69 (63–74)	mCRPC	GS 6–7 = 4 GS 8–10 = 27 missing = 2	Median (IQR): 2.5 (1.3–14.3)	NR	ADT: 33 ARPI: 33 Taxanes: 11
Xu et al. (17)	145	June 2018 – June 2021	Mean ± SD: 68.5 ± 6.6 Median (IQR): 69.0 (64.0–73.0)	BCR	GS 6 = 7 GS 7 = 72 GS 8 = 38 GS 9 = 28	Mean ± SD: 3.6 ± 0.9 Median (IQR): 0.87 (0.50–2.31)	Mean ± SD: 8.4 ± 6.1 Median (IQR): 5.8 (3.0–9.1)	RP: 114 RP + RT or ADT: 31
Kim et al. (18)	42	June 2019 – January 2021	Median (range): 70 (50–85)	Primary Staging	GS 6 = 4 GS 7a = 6 GS 7b = 9 GS 8 = 14 GS 9–10 = 9	Median (range): 11 (3.02–265.1)	NR	None: 42
Malaspina et al. (19)	25	NR	Median (IQR; range): 74 (70–78; 63–84)	Primary staging (mPCa)	GS 7b = 3 GS 8 = 4 GS 9–10 = 18	Median (IQR; range): 49 (33–140; 15–5000)	NR	ADT: 25
Pan et al. (20)	74	April 2019 – October 2020	Median (range): 67 (47–84)	BCR; early PSA progression on ADT (PSA ≤ 2 ng/mL; nmPCa)	GS 6–7 = 19 GS 8–10 = 52 Missing = 3	Median (range): 0.59 (0.17–1.98)	≤ 6 = 44 > 6 ≤10 = 23 > 10 = 7	RP + ADT: 43 RT + ADT: 1 RP + RT + ADT: 30
Chen et al. (21)	56	May 2018 – February 2021	Mean ± SD: 69.6 ± 7.0 Median (IQR): 70.0 (63.0–75.0)	CRPC	GS 6 = 4 GS 7 = 20 GS 8 = 9 GS 9 = 23	Mean ± SD: 12.5 ± 4.6 Median (IQR): 5.0 (1.5–14.5)	Mean ± SD: 7.9 ± 6.4 Median (IQR): 5.8 (3.3–9.6)	RP + ADT: 56
Fourquet et al. (22)	30	June 2017 - February 2020	Median (range): 67.8 (51–84)	HSPC: 41 CRPC: 20 (mPCa)	GS ≤ 6 = 5 GS 7a = 6 GS 7b = 9 GS 8 = 15 GS 9-10 = 25 Not available = 1	Median (range): 9.97 (0.02–7270.8)	Median (range): 5.1 (0.7–81.7)	None: 34 ADT: 18 ADT + other: 3 other: 6
Shi et al. (23)	120	July 2018 - August 2019	NR	PCa	NR	NR	NR	NR
Shi et al. (24)	138	June 2018 - December 2019	Mean ± SD: 69.2 ± 7.4	BRC: 73 Primary staging: 65	GS 6 = 4 GS 7 = 69 GS 8 = 31 GS 9 = 31 GS 10 = 3	Median (IQR) in primary staging: 56.4 (18.5–99.7) Median (IQR) in BCR: 1.1 (0.5–4.1)	NR	None: 65 Other: 73
Zhou et al. (25)	21	NR	Median (range): 66 (50–82)	Primary staging	GS 7a = 2 GS 7b = 3 GS 8 = 4 GS 9 = 5 GS 10 = 2 Not available = 5	Mean ± SD: 74.7 ± 69.2 Median (range): 41.2 (5–200)	NR	None: 21
Chen et al. (26)	72	June 2018 - August 2020	Mean ± SD: 66.4 ± 6.5 Median (IQR): 66.0 (61.0–70.8)	BCR	GS 6 = 1 GS 7 = 39 GS 8 = 18 GS 9 = 12 GS 10 = 2	Mean ± SD: 3.4 ± 1.1 Median (IQR): 0.5 (0.3–3.5)	Mean ± SD: 8.0 ± 6.5 Median (IQR): 5.9 (3.4–9.5)	RP: 34 RP + ADT: 27 RP + CHT: 4 RP + RT: 7
Wang et al. (27)	37	NR	Median (IQR): 67 (65–73)	early PSA progression on ADT (PSA ≤ 2 ng/mL; nmPCa)	GS ≤ 7 = 10 GS 8–10 = 27	Median (IQR): 3.34 (1.73–6.5)	Median (range): 3.34 (1.73–6.50)	RP + ADT: 29 RP + RT + ADT: 8

ADT, androgen deprivation therapy; ARPI, androgen-receptor pathway inhibitor; BCR, biochemical recurrence; CHT, chemotherapy; CRPC, castration-resistant prostate cancer; CT, computed tomography; GS, Gleason score (according to information reported in the included studies); HSPC, hormone-sensitive prostate cancer; mPCa, metastatic prostate cancer; nmPCa, non-metastatic prostate cancer; NR, not reported; PARP–I, PolyADP-ribose polymerase inhibitor; PCa, prostate cancer; PSA, prostate-specific antigen; PSA dt, PSA doubling time; RP, radical prostatectomy; RT, radiotherapy.

**TABLE 3:** Index test key characteristics.

References	PET tracer	Two-scan interval (days)	Administered activity	Uptake Time (minutes)	Scan time per bed (minutes)	Image analysis	Image evaluators	Other PET tracer beyond [^18^F]FDG and PSMA ligands
Pouliot et al. (14)	[^18^F]FDG [^68^Ga]Ga-PSMA-617	≤ 10	[^18^F]FDG: NR [^68^Ga]Ga-PSMA-617: 1.8-2.2 MBq/Kg (maximum 300 MBq)	[^18^F]FDG: 60 ± 5 [^68^Ga]Ga-PSMA-617: 60 ± 5	NR	Qualitative; Semiquantitative (SUVmax and others)	1 central reviewer (for ambiguous cases, a consensus was reached between the 2 central readers)	[^68^Ga]Ga-DOTATATE
Pabst et al. (15)	[^18^F]FDG [^68^Ga]Ga- PSMA-11/[^18^F]F-PSMA-1007	≤ 48 Median 5 (range 1-48)	[^18^F]FDG: Median 326 MBq (IQR: 75.7) [^68^Ga]Ga-PSMA-11: Median 96.5 MBq (IQR: 25.8) [^18^F]F-PSMA-1007: median 307 MBq (IQR: 62.3)	[^18^F]FDG: Median 69.5 (IQR: 17.3) [^68^Ga]Ga- PSMA-11: Median 42 (IQR: 10) [^18^F]F-PSMA-1007: median 83 (IQR: 4)	NR	Qualitative; Semiquantitative (SUVmax and others)	2 independent, blinded nuclear medicine physicians	[^68^Ga]Ga-FAPI-46
Pan et al. (16)	[^18^F]FDG [^68^Ga]Ga-PSMA	< 5	NR	NR	NR	Qualitative; Semiquantitative (SUVmax)	3 experienced nuclear medicine specialists	None
Xu et al. (17)	[^18^F]FDG [^68^Ga]Ga-PSMA-11	< 14	[^18^F]FDG: 3.7 MBq/Kg [^68^Ga]Ga-PSMA-11: 1.85 MBq/Kg	[^18^F]FDG: 60 [^68^Ga]Ga-PSMA-11: 50-60	3	Qualitative; Semiquantitative (SUVmax and others)	2 nuclear medicine physicians (8 and 12 y experience)	None
Kim et al. (18)	[^18^F]FDG [^18^F]F-PSMA-1007	< 7	[^18^F]FDG: 5.5 MBq/Kg [^18^F]PSMA-1007: 250 MBq	[^18^F]FDG: 60 [^18^F]F-PSMA-1007: 90	2.5	Qualitative; Semiquantitative (SUVmax)	2 nuclear medicine physicians (27 and 7 y experience)	None
Malaspina et al. (19)	[^18^F]FDG [^18^F]F-PSMA-1007	< 7	[^18^F]FDG: Median 368 MBq (IQR 333-381; range 278-398) [^18^F]PSMA-1007: Median 255 MBq (IQR 251-259; range 241-278)	[^18^F]FDG: 60 [^18^F]F-PSMA-1007: 50	2	Qualitative; Semiquantitative (SUVmax and others)	1 nuclear medicine physician (S. M.)	None
Pan et al. (20)	[^18^F]FDG [^68^Ga]Ga-PSMA-11	< 5	[^18^F]FDG: 3.7 MBq/Kg [^68^Ga]Ga-PSMA-11: 2 MBq/Kg	[^18^F]FDG: 60 [^68^Ga]Ga-PSMA-11: 60	NR	Qualitative; Semiquantitative (SUVmax)	3 nuclear medicine specialists	None
Chen et al. (21)	[^18^F]FDG [^68^Ga]Ga-PSMA-11	< 14	[^18^F]FDG: 3.7 MBq/kg [^68^Ga]Ga-PSMA-11: 1.85 MBq/kg	[^18^F]FDG: 60 [^68^Ga]Ga-PSMA-11: 50-60	3	Qualitative; Semiquantitative (SUVmax)	2 nuclear medicine physicians (8 and 12 y experience)	None
Fourquet et al. (22)	[^18^F]FDG [^18^F]DCFPyL	≤ 33	[^18^F]FDG: Mean 377.0 MBq (range 327.3-433.7) [^18^F]DCFPyL: Mean 291.3 MBq (range 221.4-399.7)	[^18^F]FDG: Mean 61.4 ± 4.6 [^18^F]DCFPyL: Mean 121.7 ± 7.9	NR	Qualitative; Semiquantitative (SUVmax; TV; TLU; TB)	3 nuclear medicine physicians	[^18^F]NaF
Shi et al. (23)	[^18^F]FDG [^68^Ga]Ga-PSMA-11	NR	[^18^F]FDG: 3.7 MBq/kg [^68^Ga]Ga-PSMA-11: 1.85 MBq/kg	[^18^F]FDG: 60 [^68^Ga]Ga-PSMA-11: 55	3	Qualitative; Semiquantitative (SUVmax)	2 nuclear medicine physicians (8 and 10 y experience)	[^11^C]C-choline
Shi et al. (24)	[^18^F]FDG [^68^Ga]Ga-PSMA-11	< 14	NR	NR	NR	Qualitative; Semiquantitative (SUVmax)	2 nuclear medicine physicians (8 and 10 y experience)	None
Zhou et al. (25)	[^18^F]FDG [^18^F]F-PSMA-1007	Median 6.5 (range 1-34)	[^18^F]FDG: Mean 388 ± 55 MBq (range 281-503) [^18^F]PSMA-1007: Mean 348 ± 52 MBq (range 266-458)	[^18^F]FDG: 60 [^18^F]PSMA-1007: 180	2	Qualitative; Semiquantitative (SUVmax; SUVmean; VOI; TBR)	2 nuclear medicine physicians	None
Chen et al. (26)	[^18^F]FDG [^68^Ga]Ga-PSMA-11	< 14	[^18^F]FDG: 3.7 MBq/kg [^68^Ga]Ga-PSMA-11: 1.85 MBq/kg	[^18^F]FDG: 60 [^68^Ga]Ga-PSMA-11: 55	3	Qualitative; Semiquantitative (SUVmax)	2 nuclear medicine physicians (8 and 20 y experience)	None
Wang et al. (27)	[^18^F]FDG [^68^Ga]Ga-PSMA-11	< 5	[^18^F]FDG: 3.7 MBq/Kg [^68^Ga]Ga-PSMA-11: 2 MBq/Kg	[^18^F]FDG: 60 [^68^Ga]Ga-PSMA-11: 60	NR	Qualitative; Semiquantitative (SUVmax)	3 nuclear medicine specialists	None

CT, computed tomography; FDG, fluorodeoxyglucose; NR, not reported; PET, positron emission tomography; PSMA, prostate-specific membrane antigen; SUV, standard uptake value; TB, total tumor burden; TBR, tumor-to-background ratio; TLU, total lesion uptake; TV, tumor volume; VOI, volumes of interest.

The number of patients per study ranges from a minimum of 10 to a maximum of 145, as shown in Table 2. Patients were enrolled in the studies between June 2017 and November 2021, aged 50 to 86 years. At the time of diagnostic imaging, patients were in the following clinical settings: primary PCa staging (3/14), mCRPC (4/14), biochemical recurrence (BCR) (3/14), primary staging + BCR (1/14), hormone-sensitive prostate cancer (HSPC) + CRPC (1/14), early PSA progression on androgen-deprivation therapy (ADT) (1/14), and PCa (1/14). In 13 out of 14 publications, information on Gleason score (GS), PSA levels, and prior treatments is provided, as detailed in Table 2.

As shown in the index test key characteristics in Table 3, in addition to [^18^F]FDG used in all studies, the PSMA ligands employed were [^68^Ga]Ga-PSMA-11 in 7/14 studies, [^18^F]F-PSMA-1007 in 3/14, [^18^F]DCFPyL in 1/14, [^68^Ga]Ga-PSMA-617 in 1/14, [^68^Ga]Ga-PSMA-11 + [^18^F]F-PSMA-1007 in 1/14, and [^68^Ga]Ga-PSMA in 1/14. The interval between the two acquisitions, PET/CT with [^18^F]FDG and PSMA ligand, was reported in 13 of the 14 studies, ranging from 1 to 48 days. The administered activity for each tracer was detailed in 12 of the 14 studies. Comprehensive data, including the time between radiotracer administration and acquisition, as well as additional acquisition and image analysis parameters, are presented in Table 3. Notably, 4 out of 14 studies also employed a third radiotracer in addition to [^18^F]FDG and PSMA ligand. However, the results of these additional tracers were excluded from this review as they fall outside the scope of its focus.

### 3.3 Risk of bias and applicability

According to the NIH quality assessment tool, none of the included studies had critical low quality.

### 3.4 Results of individual studies

#### 3.4.1 Lesions identified exclusively with [^18^F]FDG (FDG+/PSMA−)

A comprehensive analysis of the studies under review and data extraction from each revealed that, in most studies, certain lesions were positive only on [^18^F]FDG PET/CT and negative on PSMA ligand PET/CT. Further details regarding the added diagnostic value of [^18^F]FDG PET/CT in patients with prostate cancer are presented in Table 2.

#### 3.4.2 Correlation between GS, PSA levels, and [^18^F]FDG PET/CT positivity

An evaluation of the included studies demonstrated a significant correlation between GS, PSA levels, and [^18^F]FDG PET/CT-positive lesions in patients with PCa across different disease stages. Specifically, this association was evident for GS ≥ 9 according to Xu et al. (17), for GS ≥ 8 according to the first study by Chen et al. (26), and for GS ≥ 8 and PSA ≥ 7.9 ng/mL in the second study by Chen et al. (21). The clinical characteristics of patients in different studies and additional reports supporting this correlation are detailed in Tables 2, 4.

**TABLE 4:** Outcomes of the included studies.

References	Validation of PET findings	Detection rate of FDG PET compared to PSMA PET	Correlation of FDG PET findings with GS	Correlation of FDG PET findings with PSA	Additional diagnostic value by using FDG PET	Main findings
Pouliot F. et al. (14)	NR	NR	NR	NR	At least 1 [^18^F]FDG+/[^18^F]PSMA- lesion was found in 45 patients (45.9%)	IIH was observed in 81 patients (82.7%), and at least 1 [^18^F]FDG+/[^18^F]PSMA- lesion was found in 45 patients (45.9%). IIH was associated with shorter overall survival.
Pabst et al. (15)	NR	Lower	NR	NR	In a per-lesion-based analysis, 6 lesions were only [^18^F]FDG positive	Through whole-body imaging, considerable inter- and intra-patient heterogeneity of mCRPC and potential imaging phenotypes was identified.
Pan et al. (16)	NR	NR	NR	NR	[^68^Ga]Ga-PSMA-/[^18^F]FDG + lesions were observed in 7/33 (21.2%) and 8/33 (24.2%) patients at baseline and week 13, respectively.	Interlesional response heterogeneity on both baseline + week 13 [^68^Ga]Ga-PSMA and [^18^F]FDG PET/CT strongly associated with conventional PFS.
Xu et al. (17)	Composite validation, including histopathology, PSA decreases after PET-directed radiotherapy, and follow-up imaging to verify these positive results.	Lower (GS 6-8) Similar (GS 9)	Yes	Yes	No	[^18^F]FDG PET is not inferior to PSMA PET for detecting BCR with a Gleason score of 9, and [^18^F]FDG PET/CT can be considered in BCR patients with a Gleason score of 9.
Kim. et al. (18)	Intraprostatic findings were validated using MRI and histological data. Follow-up imaging was performed to confirm the presence of metastatic lesions that could not be confirmed histopathologically.	Lower	Yes	Yes	No	Tumors with [^18^F]FDG uptake are associated with larger size, a ductal-dominant type, and are likely to undergo metastasis at staging and biochemical failure postoperatively. Adding [^18^F]FDG PET/CT to [^18^F]PSMA PET/CT can help identify tumors with aggressive biology.
Malaspina et al. (19)	Absence of histological verification of potential metastases. Only PSMA uptakes with corresponding findings on CT (sclerotic or lytic lesion) were included in the analysis.	Lower	NR	NR	NR	A heterogeneous increase in [^18^F]F-PSMA uptake after ADT was detected, most evidently in bone metastases. A negative correlation between the PSMA flare and the intensity of glucose uptake and the decrease of serum PSA suggests that lesions presenting with such flare might potentially be less aggressive.
Pan et al. (20)	NR	NR	NR	NR	23% (17/74) of the patients had at least one discordant [^18^F]FDG-avid lesion	Dual-tracer PET/CT-guided SBRT delivered superior local control rates in comparison to ADT alone and had minimal toxicity.
Chen et al. (21)	Composite validation: Among the 13 CRPC patients with [^68^Ga]Ga- PSMA-, [^18^F]FDG + lesions, 2 were verified by histopathology, 2 by decreasing PSA levels after radiotherapy, and 9 by imaging.	Lower	Yes	Yes	Of the 169 lesions detected in 48 patients, 34 lesions were [^68^Ga]Ga- PSMA-, [^18^F]FDG + .	CRPC patients with a high Gleason score (≥ 8) and a high PSA (≥ 7.9 ng/mL) may benefit from [^18^F]FDG PET/CT. For staging, the addition of [^18^F]FDG PET/CT could increase the detection rate of local recurrence, lymph node metastasis, distant metastasis, and any location from 14.3 to 19.6%, from 42.9 to 55.4%, from 35.7 to 39.3%, and from 75.0 to 85.7%, respectively, when compared with [^68^Ga]Ga-PSMA PET/CT alone.
Fourquet et al. (22)	A biopsy was performed in 17 men who underwent [^18^F]FDG, revealing 3 false-negatives and 15 true-positives for the PET tracer.	Lower	NR	Yes	Among the 322 lesions detected by [^18^F]FDG or [^18^F]DCFPyL, 68 were concordant, 232 were detected by [^18^F]DCFPyL only, and 22 were detected by [^18^F]FDG only.	Further research is warranted to elucidate the utility of [^18^F]FDG PET as prognostic tools and complementary agents to [^18^F]DCFPyL in understanding tumor heterogeneity patterns in PCa metastases.
Shi et al. (23)	NR	NR	NR	NR	NR	[^68^Ga]Ga-PSMA and [^11^C]choline uptake in ganglia was common, and [^18^F]FDG-positive ganglia were observed at lower frequency. Using [^68^Ga]Ga-PSMA, [^11^C]choline and [^18^F]FDG uptake and anatomic location and configuration, the differentiation of ganglia from adjacent LNM is feasible.
Shi et al. (24)	NR	NR	No	No	NR	The [^18^F]FDG-PET and [^68^Ga]Ga-PSMA-11-PET SUVmax, especially when combined, could well differentiate LNM from ganglia.
Zhou et al. (25)	NR	Lower	NR	NR	NR	[^18^F]F-PSMA-1007 showed superiority over [^18^F]FDG because its high detecting rate of PCa lesions and excellent tumor uptake.
Chen et al. (26)	Of the 12 BCR patients with [^18^F]FDG + findings, 3 patients were confirmed by histopathology, 4 patients by imaging, and 5 patients by the decline in PSA level after treatment. All the [^18^F]FDG + findings were confirmed as true positive.	NR	Yes	Yes	The detection rate of [^18^F]FDG PET/CT was 16.7% (12/72) in BCR patients with [^68^Ga]Ga-PSMA-negative findings.	PSA level and Gleason score are independent predictors of [^18^F]FDG-positive findings, and BCR patients with [^68^Ga]Ga-PSMA-negative findings with high PSA and Gleason score (≥ 8) are most likely to benefit from [^18^F]FDG PET/CT.
Wang et al. (27)	Except for non-compliance in 1 of the 9 patients with [^68^Ga]Ga-PSMA-11(-)/[^18^F]FDG(+) lesions, patients were confirmed with composite validation (histopathology, response or progression on imaging and decline in PSA level after therapy) with high true positive rate (8/9).	Lower	Yes	Yes	Of the 114 lesions detected in 29 patients, 81 were [^68^Ga]Ga-PSMA-11(+)/[^18^F]FDG(± ) and 33 lesions were [^68^Ga]Ga-PSMA-11(-)/[^18^F]FDG(+)	Using [^68^Ga]Ga-PSMA and [^18^F]FDG PET, a high prevalence of N + /M + disease and a significant proportion of [^68^Ga]Ga-PSMA-11(-)/[^18^F]FDG(+) disease was observed in patients with an early PSA progression during castration.

ADT, androgen deprivation therapy; BCR, biochemical recurrence; CRPC: castration-resistant prostate cancer; CT, computed tomography; DCFPyL, piflufolastat; FDG, fluorodeoxyglucose; GS, Gleason score; IIH, intrapatient intermetastatic heterogeneity; LNM, lymph node metastasis; MRI, magnetic resonance imaging; NR, not reported; PCa, prostate cancer PET, positron emission tomography; PSMA, prostate-specific membrane antigen; PSA, prostate spacific antigen; SBRT, stereotactic body radiation therapy.

#### 3.4.3 Prognostic significance

The prognostic role of integrating [^18^F]FDG PET/CT into the diagnostic pathway for patients with PCa was thoroughly evaluated by Kim et al. (18), who concluded that the addition of [^18^F]FDG PET/CT to PSMA ligand PET/CT can help identify tumors with aggressive biological behavior.

## 4 Discussion

PSMA-ligand PET/CT is widely used for staging and biochemical recurrence of PCa. However, PCa may appear negative on PSMA-ligand PET/CT (28, 29), and, as observed in the studies analyzed in this review, low PSMA expression could be associated with [^18^F]FDG positivity.

An analysis of the available data confirms that FDG+/PSMA− lesions are not uncommon in PCa and can be observed in a significant proportion of patients in different settings (14, 20, 21). Additionally, a subgroup of mCRPCa patients, accounting for 6% in the study by Pouliot et al., exhibited exclusively FDG+/PSMA− disease (i.e., absence of PSMA + disease), with a similar prevalence to the 10% reported in the TheraP trial (30).

For these patients, [^18^F]FDG PET/CT could serve as a useful complementary diagnostic tool at various stages of prostate cancer management, given that, until now, [^18^F]FDG positivity has primarily been considered at a late stage of the disease as a selection criterion before radioligand therapy or as a poor prognostic factor following such treatments (31).

However, the utility of [^18^F]FDG PET/CT may extend to the staging phase, as demonstrated in the study by Kim et al., which also evaluated its prognostic impact (18). The collected data indicated that PCa exhibiting [^18^F]FDG uptake were significantly larger, associated with ductal-dominant histology, and characterized by higher GS and initial PSA levels compared to those [^18^F]FDG negative (18). Similarly, Chen et al. found that patients with FDG+/PSMA− lesions had higher GS and PSA levels than those without such lesions (21).

Of particular relevance is the observation that patients with [^18^F]FDG-avid tumors had a higher incidence of lymph node and/or distant metastases at initial staging, as well as an increased biochemical recurrence rate postoperatively, compared to those with non-[^18^F]FDG-avid tumors (18).

Using a dual-tracer approach could enable more accurate diagnostic assessment in a specific subgroup of patients. Furthermore, these data suggest that identifying this patient subgroup could have significant prognostic implications. However, as reported in the study by Chen et al. (21), only 23.2% of the analyzed patients had at least one FDG+/PSMA− lesion, underscoring the need for careful patient selection for [^18^F]FDG PET/CT. The objective is to optimize its use, reserving it for cases where it provides a clear diagnostic benefit, particularly in high-risk PCa patients.

An analysis of the studies suggests that the GS may serve as a valuable criterion for predicting the presence of FDG+/PSMA− lesions. A recurring finding across the reviewed studies was that patients with a high GS were more likely to present FDG+/PSMA− lesions than those with a lower score. Specifically, Xu et al. highlighted this correlation for a GS ≥ 9 (17), while both studies by Chen et al. found an association starting from a GS of 8 (21, 26).

Previous studies have also confirmed the diagnostic value of [^18^F]FDG PET/CT in patients with PCa and high GS (9, 32). In line with this evidence, our analysis suggests that the GS is a key predictor for identifying patients with FDG+/PSMA− lesions. Supporting this finding, Chen et al. demonstrated that the probability of detecting FDG+/PSMA− lesions was 0% in patients with a GS < 8, while it reached 61.5% in those with a GS ≥ 8. Adding [^18^F]FDG PET/CT in the low-score group did not improve the detection rate compared to PSMA-ligand PET/CT alone. Conversely, in patients with high GS, integrating [^18^F]FDG PET/CT increased the detection rates of local recurrence, lymph node metastases, and distant metastases from 0 to 7.7%, 30.8 to 61.5%, and 53.8 to 61.5%, respectively, compared to PSMA-ligand PET/CT alone (21). These findings suggest that [^18^F]FDG PET/CT may not be indicated in patients with a low probability of FDG+/PSMA− lesions but could be more beneficial in those with a high likelihood of such lesions.

While the correlation between a GS ≥ 9 and the presence of FDG+/PSMA− lesions appears well established (17), the association with a GS of 8 requires further investigation. Moreover, the implications of these findings will help in selecting patients for radionuclide therapy, as a prerequisite for this is that PCa lesions are PSMA + (33).

Although studies with therapeutic intent were excluded from the present systematic review, several pivotal clinical trials that employed dual-tracer PET imaging for patient selection in [^177^Lu]Lu—PSMA radioligand therapy deserve detailed discussion. In the TheraP trial, involving patients with mCRPC who had previously received docetaxel, [^18^F]FDG PET/CT was systematically used during screening to identify and exclude individuals with discordant disease, and approximately 18% of screened patients were excluded due to this finding (30). Similarly, the ENZA-p trial, which randomized patients with treatment-naïve mCRPC to enzalutamide ± [^177^Lu]Lu-PSMA-617, incorporated [^18^F]FDG PET/CT at baseline to refine patient selection. While the exclusion criteria in this study were less stringent than in TheraP, the use of [^18^F]FDG PET/CT remained a central component in identifying those with low or heterogeneous PSMA expression, who may be less likely to benefit from PSMA-targeted therapy (34). Moreover, the UpFrontPSMA trial extended this approach into an earlier disease setting, evaluating [^177^Lu]Lu-PSMA-617 followed by docetaxel versus docetaxel alone in patients with *de novo* high-volume metastatic hormone-sensitive prostate cancer (35). [^18^F]FDG PET/CT was used at baseline to exclude patients with extensive FDG+/PSMA− disease, highlighting its growing role in the late-stage setting and in therapeutic stratification during initial management. These studies underscore a paradigm shift whereby [^18^F]FDG PET/CT is no longer viewed solely as a diagnostic adjunct but increasingly as a biological stratification tool. However, as confirmed by our systematic review, identifying patients with FDG+/PSMA− mismatch disease remains a substantial unmet clinical need, and more studies are needed to establish predictive factors for this condition. While guidelines and expert consensus frequently recommend [^18^F]FDG PET/CT “only in selected cases”, precise criteria, whether clinical, biochemical, or imaging-based, are largely undefined. Recent efforts have been made to address this gap. For example, Pan et al. developed a prediction nomogram based on clinical and imaging features (including SUVmax, PSA levels, bone metastases, prior docetaxel treatment, and alkaline phosphatase), achieving an AUC of 0.83 for the model and demonstrating its potential to guide the use of [^18^F]FDG PET/CT more selectively in mCRPC (36). Furthermore, a study by Telli et al. has shown that the [^18^F]FDG-derived tumor burden is an independent prognostic factor for overall survival, even among patients eligible for PSMA-based radioligand therapy, regardless of treatment received. This finding highlights the prognostic value of [^18^F]FDG PET/CT beyond simple eligibility determination and supports its role in treatment planning (37). Notably, predictive factors for FDG+/PSMA− disease may vary depending on the phase of PCa’s natural history. GS (particularly ≥ 8) appears to be a significant predictor in the primary staging setting. In contrast, in advanced mCRPC, disease biologic behavior may be influenced by prior systemic therapies and treatment-induced neuroendocrine differentiation, potentially diminishing the relevance of the original histological grading.

However, it is noteworthy that studies employing dual-tracer PET imaging for the purpose of selecting patients for [^177^Lu]-PSMA radioligand therapy were excluded from this systematic review. These studies were not considered eligible (as stated in the methods section) as the primary aim of our review was to explore the diagnostic potential of [^18^F]FDG PET/CT in PCa patients across different clinical settings, regardless of pre-therapeutic stratification. Therefore, in order to maintain methodological consistency and ensure alignment with the objectives of this review, such studies were excluded from the analysis.

Finally, it should be underlined that our systematic review has several limitations. The most important are the limited number of available studies on the topic of interest and the significant heterogeneity of the clinical characteristics of patients enrolled (including different PCa settings) and methodological aspects of eligible studies. Due to this heterogeneity a meta-analysis is not appropriate (12). Another consideration emerging from the literature is the non-negligible incidence of false-positive bone findings on PSMA-ligand PET/CT scans. The occurrence of unspecific bone uptakes varies considerably depending on the PSMA tracer used, with [^18^F]PSMA-1007 being associated with the highest prevalence (38). Consequently, the actual number of PSMA-positive bone lesions that truly represent metastatic deposits may be overestimated, particularly in the studies employing [^18^F]PSMA-1007 (15, 18, 19, 25).

Notably, we have not considered the specific diagnostic accuracy values of [^18^F]FDG PET/CT and PSMA—ligand PET/CT scans in PCa. The current evidences already showed that PSMA—ligand PET/CT had higher diagnostic accuracy in detecting PCa lesions compared with [^18^F]FDG PET/CT both in terms of sensitivity and specificity (39). Results from a previous meta-analysis already showed that the pooled sensitivities of PSMA—ligand PET/CT ranged from 91 to 92% compared to 75% for [^18^F]FDG PET/CT; the pooled specificity ranged from 73 to 88% for PSMA—ligand PET/CT compared to 64% for [^18^F]FDG PET/CT (39).

As dosimetric aspects are relevant for nuclear medicine imaging and therapy of PCa (40), we would like to underline that performing dual-tracer PET/CT will increase the radiation exposure for the patients. Future studies should better clarify the advantages of performing dual-tracer PET/CT compared to the potential risks related to an increased radiation exposure. Several methods could be used to reduce the radiation exposure related to the PET and CT component of hybrid imaging (41, 42).

Even taking into account these limitations, we believe that our evidence-based manuscript has provided significant information about dual-tracer PET/CT in PCa. In particular, compared to a previous systematic review on the same topic (43), we have included more studies excluding case reports (affected by significant biases) providing an update of the literature through a systematic summary and suggesting further studies to solve the current knowledge gaps. As practical application, we can suggest the use of dual-tracer PET/CT in selected cases of PCa, in particular in aggressive variants, but this approach should be confirmed by further well-designed studies.

## 5 Conclusion

In conclusion, our systematic review demonstrated that dual-tracer PET/CT approach improved PCa lesion detection due to [^18^F]FDG-positive/PSMA-negative (FDG+/PSMA−) lesions in a subset of PCa patients, particularly those with GS ≥ 9. However, in patients with GS < 8, [^18^F]FDG PET/CT did not significantly improve lesion detection over PSMA ligand PET/CT alone. The presence of FDG+/PSMA− lesions correlated with aggressive tumor biology, increased risk of metastases, and worse prognosis.

Available literature data suggest that adding [^18^F]FDG PET/CT to PSMA-ligand PET/CT may have a role in PCa in selected patients, particularly those with aggressive variants of PCa. Although the potential added value of dual-tracer PET/CT for PCa lesion detection in this specific patient group has been established, the prognostic impact and cost-effectiveness of dual-tracer PET/CT in PCa remain to be determined through further studies.

## Data Availability

The original contributions presented in the study are included in the article/supplementary material, further inquiries can be directed to the corresponding author.
